# Sonification of Arm Movements in Stroke Rehabilitation – A Novel Approach in Neurologic Music Therapy

**DOI:** 10.3389/fneur.2016.00106

**Published:** 2016-06-30

**Authors:** Daniel S. Scholz, Sönke Rohde, Nikou Nikmaram, Hans-Peter Brückner, Michael Großbach, Jens D. Rollnik, Eckart O. Altenmüller

**Affiliations:** ^1^Institute of Music Physiology and Musicians’ Medicine, University of Music, Drama and Media, Hannover, Germany; ^2^Institute for Neurorehabilitational Research (InFo), BDH-Clinic Hessisch Oldendorf, Teaching Hospital of Hannover Medical School (MHH), Hessisch Oldendorf, Germany

**Keywords:** sonification, stroke, neurorehabilitation, neuroplasticity, music-supported therapy

## Abstract

Gross motor impairments are common after stroke, but efficient and motivating therapies for these impairments are scarce. We present an innovative musical sonification therapy, especially designed to retrain patients’ gross motor functions. Sonification should motivate patients and provide additional sensory input informing about relative limb position. Twenty-five stroke patients were included in a clinical pre–post study and took part in the sonification training. The patients’ upper extremity functions, their psychological states, and their arm movement smoothness were assessed pre and post training. Patients were randomly assigned to either of two groups. Both groups received an average of 10 days (M = 9.88; SD = 2.03; 30 min/day) of musical sonification therapy [music group (MG)] or a sham sonification movement training [control group (CG)], respectively. The only difference between the two protocols was that in the CG no sound was played back during training. In the beginning, patients explored the acoustic effects of their arm movements in space. At the end of the training, the patients played simple melodies by coordinated arm movements. The 15 patients in the MG showed significantly reduced joint pain (*F* = 19.96, *p* < 0.001) in the Fugl–Meyer assessment after training. They also reported a trend to have improved hand function in the stroke impact scale as compared to the CG. Movement smoothness at day 1, day 5, and the last day of the intervention was compared in MG patients and found to be significantly better after the therapy. Taken together, musical sonification may be a promising therapy for motor impairments after stroke, but further research is required since estimated effect sizes point to moderate treatment outcomes.

## Introduction

Stroke is a major cause of mortality and morbidity in both the developed and developing world (1). In Germany, stroke is one of the most common disorders with an estimated 200,000 first events and 66,000 recurrent events in 2008 (2). The World Health Organization stresses the need to collect high quality longitudinal data on rehabilitation and to improve the comparability between studies (3).

The rehabilitation of stroke patients remains a challenge, although there are currently several new training programs under development that aim at improved efficiency and sustainability of stroke rehabilitation (4). Some of the traditional rehabilitation programs lack general acceptance by patients, due to the required endurance and high demands on the patients’ cooperation, which sometimes is perceived as a frustrating experience (5). Yet, even the well-established standard physiotherapies do not unambiguously provide evidence of efficacy when it comes to improvement of skilled motor behavior (6–8). Therefore, there is an urgent need for innovative, motivating, and goal-directed training protocols in stroke rehabilitation.

In this article, we present an innovative approach to rehabilitation by retraining the gross motor functions of the affected upper limbs using musical sonification. In an earlier clinical feasibility study (9), we showed how a musical sonification therapy could be applied. The data presented herein were obtained with this method from a larger number of patients. Sonification stands for the usage of non-speech sound representing otherwise not audible information (10). One of the first sonification devices was the Geiger–Müller counter, which detects electromagnetic radiation and communicates a decay by a click sound. In the present study, arm movements were translated into sound. In two earlier studies, we demonstrated the efficacy of a music-supported stroke rehabilitation training utilizing a MIDI drumset and a MIDI piano (11, 12). Stroke patients with some residual abilities to move the arm and the fingers were instructed to play simple tunes (nursery rhymes or folk songs) on either instrument. We could show that auditory sensorimotor circuits established *via* this form of music-supported therapy (MST) promotes beneficial neuroplasticity in stroke patients (13, 14). One of the few constraints of MST was that it was mainly designed to retrain fine-motor skills on MIDI instruments. And it did not provide continuous real-time feedback for the gross motor functions of the arm, which are more frequently impaired in early rehabilitation stages. A real-time movement feedback may be beneficial since it informs the patients about the way they move, not only whether they hit the target or not. With the musical sonification therapy presented here, patients repeatedly train movements with their affected arm in a predefined space. They form associations of their relative armposition in space and the corresponding sound at this specific position. At the end, they play familiar melodies by moving their arm. This musical sonification therapy, therefore, broadens the scope to train stroke patients from an earlier stage on, when still suffering from gross motor dysfunction. Musical sonification will not only contribute to the motivation of the patients due to its playful and positive emotional character, but may also improve motor control, since auditory real-time feedback of the patient’s arm movements can be substituted for potentially lost proprioception. There are several preliminary studies with healthy participants that apply non-musical sonification in motor control and the perception of movements (15–17). Schmitz et al. found that sonifying breast stroke movements led to more precise perceptual judgments of movement velocity. They showed that sonification of movements amplifies the human action observation system as indicated by more pronounced fMRI connectivity patterns between the activation peaks of the left superior and medial posterior temporal regions with the basal ganglia, the thalamus, and frontal regions for movement congruent sonification stimuli. Thus, sonification may be an important method to enhance training and therapy effects in neurological rehabilitation. Chen et al. developed a real-time, multimodal feedback system for stroke rehabilitation (18). This sonification system was tested with stroke patients and showed promising results (19). However, in their design, music was only a passive byproduct of arm movements. That means participants did not play with the sonification sound intentionally. They moved their arms and harmonic music progressions were played back to them. In contrast to that, we developed a musical sonification therapy to train stroke patients to explicitly and consciously play music through intended movements of their affected upper extremity. Thus, we hoped to be able to use the beneficial effects of music on neuroplasticity to facilitate the recovery after stroke (13). Since in other studies repetitive exercise has been shown to be effective (8, 20), our training is of a repetitive nature too. We hypothesize that the auditory cues provided by the sonification may make multimodal associative learning possible where otherwise mere visual and motor learning would have taken place. We assume that patients will benefit in their rehabilitation process from guided attention, necessary concentration, and long-term motivation to play music. Rohrer et al. (21) (see also references therein) describe an increase of several movement smoothness indices in both acute and chronic stroke patients during movement therapy. Hence, the present study additionally investigated changes in movement smoothness over the course of the therapy. After having evaluated an optimal two-dimensional sonification mapping (22), we now present a more detailed analysis of our three-dimensional musical sonification therapy with a larger sample (9).

## Materials and Methods

### Patients

Twenty-five inpatients (11 women, see Table 1 for details) at the BDH Neurological Rehabilitation Hospital in Hessisch Oldendorf, Germany, participated after giving informed consent. They suffered from a moderate impairment of motor function of the upper extremity after stroke. Inclusion criteria were (a) patients had to have residual function of the affected extremity (i.e., the ability to move the affected arm and the index finger without help from the healthy side), furthermore, (b) an overall Barthel index higher than 50 was required, and (c) patients had to be right-handed. Patients with other neurological or psychiatric disorders were excluded.

**Table 1:** **Demographic details of the 25 patients**.

	Music group	Control group
Number of subjects	15	10
Gender (m/f)	8/7	6/4
Age (M and SD) range (years)	68.8 ± 13.6 (32–86)	72.2 ± 8.4 (57–85)
Affected arm: right	15	10
Right-handed	15	10
Days after stroke (Median)	32.5	28
Barthel index (M and SD)	56.5 ± 25.3	47 ± 35.9

Patients were pseudorandomly assigned to the experimental or to the control group (CG) by the supervisor of the study who was not the experimenter. The experimental group received conventional physiotherapy plus an average of 10 days of a musical sonification training [music group (MG), henceforth].

The CG also received conventional physiotherapy plus a sham sonification movement training with exactly the same movements required as in the sonification study, but with no sound being played back. All patients were native German speakers. The study was approved by the Ethics Review Board of the Hannover Medical School (MHH).

### Evaluation of Motor Functions, Stroke Impact, and Movement Smoothness

#### Procedure

Patients were tested pre and post training with a battery of clinical motor function tests and a psychological questionnaire. The test battery consisted of (a) the upper extremity part of the Fugl–Meyer assessment (FMA), still considered the gold standard in evaluating motor recovery after stroke (23, 24). The FMA consists of four bigger subsections. FM.A–D assesses the motor function of the affected arm by checking reflexes, volitional movements, wrist and hand function, and the coordination of the upper extremity. In FM.H, the tactile sensation compared to the non-affected other extremity is assessed. In FM.I, passive joint motion is assessed and FM.J passively measures joint pain. (b) The action research arm test (ARAT) rates upper limb functioning by using observational methods and collecting behavioral data (25, 26). (c) The box and block test (BBT) assesses unilateral gross manual dexterity (27, 28). (d) The nine-hole pegboard test (NHPT) measures finger dexterity (29) and (e) the stroke impact scale (SIS) evaluates the health status following a stroke, including subscales for emotional well-being, memory, thinking, and social participation. The subscales are SIS.1 that asks for physical problems, which may have occurred as a result of the stroke; SIS.2 investigates memory and thinking of the patient; SIS.3 assesses mood and emotions; SIS.4 checks for the communication skills in speaking, reading, and writing; SIS.5 determines how impaired the patient is during daily activities; in SIS.6, the mobility of the patient is investigated; SIS.7 assesses the remaining function of the affected hand; in SIS.8, the patient is asked to which extent he or she is impaired in their social activities; SIS.9 is a self-rating of the patient on how far the stroke recovery has progressed (30, 31).

Movement data were recorded in the MG only using a custom made computer program and two inertial sensors (Xsens, X-MB-XB3), one attached to the fore arm close to the wrist and one attached to the upper arm of the patients. It took approximately 1 h to complete the test battery at the beginning and at the end of the study. Regular training sessions lasted approximately 30 min.

### Sonification Training

#### Training

**Figure 1:**
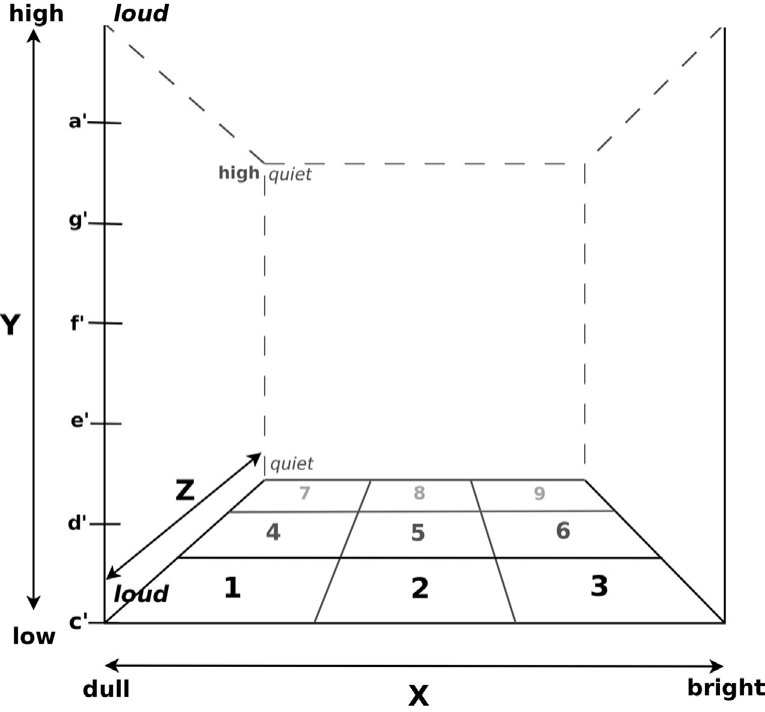
**The 3D space defined by a wooden frame**. Pitch was mapped onto the *y*-axis ranging from c′ at the bottom to a′ at the top. Brightness was mapped onto the *x*-axis from the left (dull) to the right (bright) and volume onto the *z*-axis with loudness increasing with increasing proximity. Positions in the *x*–*z* plane were labeled for ease of instruction.

After the pretests the patients received either an average of 10 days (M = 9.88; SD = 2.03) of musical sonification training (MG), or 10 days of sham sonification training (CG), following the same protocol as MG but with loudspeakers switched off. The whole procedure followed a standardized protocol to train gross motor functions of the affected right upper extremity in a repetitive manner. Patients were seated as close as possible to the desk with the wooden 3D space frame atop so that the board nearly touched their stomach. Depending on whether in a wheelchair or not, the desk with the 3D space on was adjusted to the individual needs of the patients before starting the training. To get acquainted with the sonification system and the acoustic effects produced by their own arm movements, patients first had to freely move their arm in a three-dimensional sonification space, a wooden cubic frame of 51 cm side length, confined by four vertical beams in the corners of the bottom board (Figure 1). The beams were labeled with the note pitches; the board was subdivided into nine labeled fields for ease of instructions.

Movement sonification was implemented so that upward movements resulted in an ascending C major scale from c′ (256 Hz, in Helmholtz pitch notation) to the sixth interval a′ (440 Hz). Vertical movements in this space resulted in a change in brightness of sound and, thus, mimicking real musical instrument timbres (modeled by varying the number and amplification of overtones in the sound synthesis; SynthesisToolKit – STK; Cook and Scavone (32); from a rather dull clarinet sound at the very left to saxophone in the middle and a bright sounding bowed instrument at the very right). Movements along the *z*-axis caused an increase in loudness from proximal to distal. After a first exploration phase to allow for implicitly learning the rules of the musical sonification, more complex exercises followed, demanding incremental degrees of difficulty: At the beginning of each training session patients had to play four upward and downward legato C major scales at position 1 (Figure 1). The same exercise was then repeated at positions 2, 3, 7, and 9. [You can listen to the legato scale playing of a patient at day 1 (Audio S1 in Supplementary Material), day 5 (Audio S2 in Supplementary Material), and the last training day (Audio S3 in Supplementary Material)]. These exercises were followed by playing musical intervals by moving the arm faster but as precisely as possible, from c′ to d′, from c′ to e′, from c′ to f′, from c′ to g′, and from c′ to a′. This exercise was repeated four times at position 1 and then likewise at positions 2, 3, 7, and 9. The final goal of the training was to teach patients to play several simple nursery rhymes or other familiar tunes only by moving their affected right arm in the three-dimensional sonification space.

The experimenter gave verbal instructions for the training procedure. Additionally, the experimenter pointed at the visual cues written at the positions on the wooden frame of the 3D space (Figure 1). When playing the melodies, patients could read the required “coordinates” from a sheet provided. All melodies were played vertically, i.e., along the *y-*axis, at position 1 (Figure 1). Tones could be repeated by dipping the hand horizontally in one direction while maintaining vertical position. Patients always moved their impaired arms by themselves. Arm movements were never guided nor physically supported by the experimenter.

Patients’ arm movements were sonified in real time using two small inertial sensors (Xsens, X-MB-XB3) placed at the wrist and the upper arm of the affected limb. The continuous data stream comprising acceleration, rotation, and gravity were transferred *via* Bluetooth to a laptop and stored for later evaluation in the MG only. The spatial information of the arm movements in 3D space were sonified in real time. The only difference in the training procedure for the sham sonification group (CG) was the muted playback system. Otherwise, exactly the same exercises were carried out during the training sessions.

### Data Analysis

Statistical analysis was conducted using R^1^ (version 3.2.1) in RStudio Server^2^ (version 0.99.467) on data of the motor function tests and the SIS. Motor test and questionnaire data were preprocessed and tested whether they complied with ANCOVA assumptions. Pretest scores were then used as covariate either in separate ANCOVAs for each response variable, or in the Johnson–Neyman test when applicable: when comparing two groups with respect to their performance before and after treatment, and the assumption of homogenous regression slopes is violated, the Johnson–Neyman technique is used instead of ANCOVA. It allows for the two groups to have different slopes, and tests whether they differ. Additionally, it determines an “area of significance” (33) where the two groups show a statistically meaningful difference in their posttreatment score, after controlling for the differing slopes.

Arm movement data from the four trials at position 1 (Figure 1) from each MG patient were collected and transformed into Cartesian coordinates using a custom-made computer program. Three-dimensional movement trajectories from upward and downward legato C major scales were manually selected and Butterworth lowpass filtered (cut-off 8 Hz) to eliminate tremor movements. Movement smoothness was calculated as the curvature index κ:

$$\kappa^{2}=\frac{\left({\dot{x}}^{2}+{\dot{y}}^{2}+{\dot{z}}^{2}\right)\left({\ddot{x}}^{2}+{\ddot{y}}^{2}+{\ddot{z}}^{2}\right){\left(\dot{x}\ddot{x}+\dot{y}\ddot{y}+\dot{z}\ddot{z}\right)}^{2}}{{\left({\dot{x}}^{2}+{\dot{y}}^{2}+{\dot{z}}^{2}\right)}^{3}}$$

Osu et al. (34) (see ibid. for advantages of curvature over other measures for smoothness, like jerk, or snap), representing the inverse of the movement radius for each trajectory point. To synchronize an increase in movement smoothness with an increase in κ and to account for positive skewness of κ, the median negative logarithm for each movement segment was taken.

**Figure 2:**
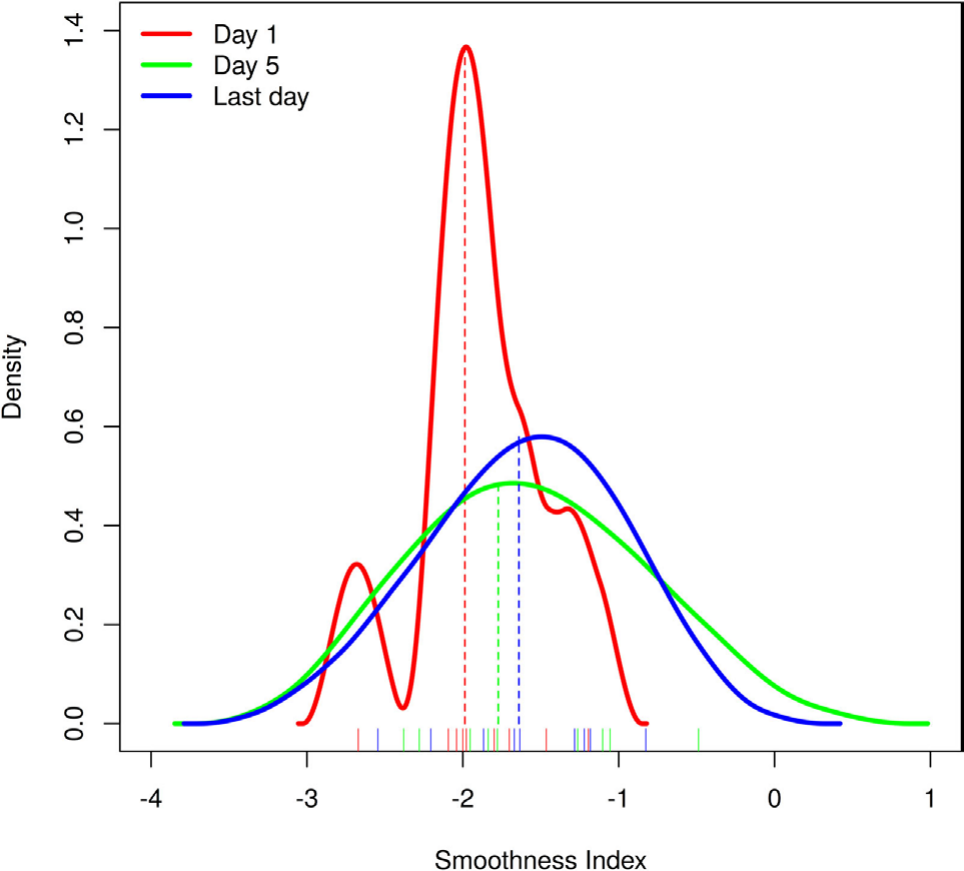
**Movement smoothness at day 1, day 5, and the last day of the intervention was compared in the treatment group (MG) patients and found to be significantly better after the therapy**. Shown here are the kernel density estimates of the MG smoothness measures at day 1 (red line), day 5 (green line), and the last day of the intervention (blue line; see Materials and Methods and Results for details) with the smoothness index shown on the *x*-axis and the density estimation on the *y*-axis. Medians of the corresponding distribution are depicted as dashed lines in corresponding color.

These values were then compared between the MGs’ first, fifth, and last therapy sessions using Friedman’s test, followed by Wilcoxon’s signed rank test as *post hoc* test to determine an increase in smoothness during (day 5) or after the end of the sonification training. The kernel density estimation of the transformed κ (see Figure 2) was calculated with a data-driven kernel suggested by Sheather and Jones (35) [cited in Ref. (36)].

## Results

### Motor Tests

**Figure 3:**
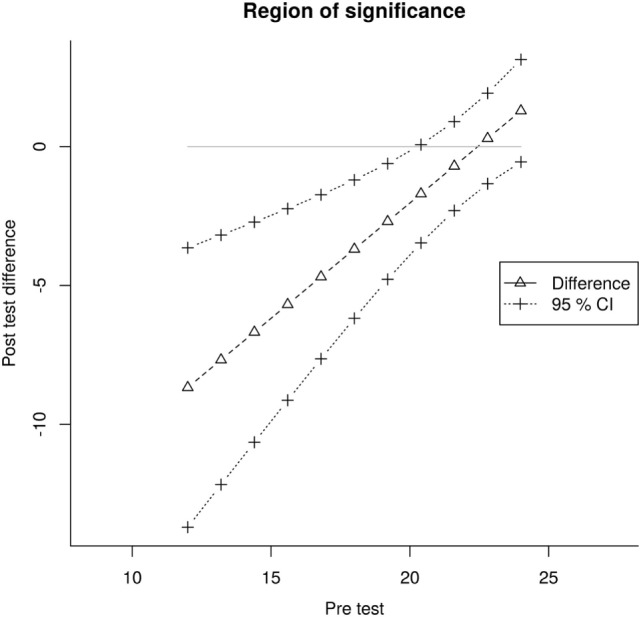
**Fugl–Meyer joint pain subscale results**. Taking into account the unequal regression slopes of CG and MG, the Johnson–Neyman technique estimates the two groups’ post-test difference (triangles, dashed straight line) from the joint pretest values and determines upper and lower confidence intervals (CI; crosses, dotted lines). The triangles and crosses do not represent individual data points but estimated values. The CIs and the solid straight line at *y* = 0, confine the area where the two groups differed significantly in their post-test Fugl–Meyer Joint pain score. Compared to CG patients, MG patients with a pretest score below 20 seemed to benefit from the additional sonification therapy.

The main results of this study are depicted in Table 2. Although the ANCOVA group comparisons for ARAT, BBT, and NHPT were non-significant, MG patients showed a significantly higher improvement compared to the CG in the subscale FM.J of the FMA [*F*(1,21) = 21.23, *p* < 0.05], as shown by the Johnson–Neyman technique, suggesting that they perceived reduced joint pain after the training. The two groups’ corrected pre–post differences differed significantly for pretest scores below 20, suggesting an advantage for the sonification therapy over sham treatment for patients scoring low in the FM.J subscale (Figure 3).

**Table 2:** **ANCOVA group comparison results for the motor tests and the stroke impact scale**.

Test (pretest: group interaction)	Sum Sq	Mean Sq	*F*	*p*
ARAT	6.635	6.635	0.1102	0.7432
BBT	0.7481	0.7481	0.01645	0.8994
NHPT	1694	1694	2.206	0.1558
FM.A–D (motor function)	0.3989	0.3989	0.01179	0.9146
FM.H (sensation)	0.8866	0.8866	1.641	0.2155
FM.I (passive joint motion)	1.775	1.775	0.363	0.5536
FM.J (joint pain)	–	–	–	see Results and Figure 2
SIS (total)	2091	2091	4.63	0.0445*
SIS.1 (physical problems)	0.7875	0.7875	0.002578	0.9601
SIS.2 (memory and thinking)	69.03	69.03	0.2237	0.6426
SIS.3 (mood and emotions)	159.3	159.3	0.4738	0.5011
SIS.4 (communication skills)	48.42	48.42	0.1189	0.7347
SIS.5 (daily activities)	168.6	168.6	0.6058	0.4477
SIS.6 (mobility)	39.39	39.39	0.1303	0.7229
SIS.7 (affected hand function)	878	878	4.278	0.0552
SIS.8 (social activities)	833.5	833.5	0.8634	0.3666
SIS.9 (stroke recovery rating)	207.4	207.4	0.8629	0.3667

*ARAT, action research arm test; BBT, box and block test; NHPT, nine-hole pegboard test; FM, Fugl–Meyer assessment; SIS, stroke impact scale*.

Significant group differences at the alpha = 0.05 – level are indicated by *.

### Stroke Impact Scale

The SIS total value was significantly higher for the MG as compared to the CG, after the training (*F* = 4.63, *p* = 0.0445). But, after correcting for the random group difference prior to the training, there was no more pretest: group interaction to be found [*F*(1,21) = 3.83, *p* > 0.05]. A pre test group difference for SIS.2 [two-sided paired *t*-test (*t* = 2.229, df = 19.92, *p* = 0.0375)] was detected. For that reason this specific subscale was not included into further evaluation. The subscale SIS.7 showed a trend (*F* = 4.278, *p* = 0.0552) toward better hand function of the MG patients after the training. All other ANCOVA group comparisons were non-significant.

### Arm Movement Smoothness

Movement data from two MG patients could not be retrieved due to technical failure of the recording system. For the remaining 13 patients, trajectory smoothness was derived at day 1, day 5, and the last day of their training. A significant difference between training days was shown with Friedman’s test (χ^2^ = 6.222, df = 2, *p* = 0.0445; see Figure 2), and Wilcoxon *post hoc* tests showed a significantly higher movement smoothness at the last day as compared to the first training day (*V* = 7, *p* = 0.037). But after applying the Bonferroni–Holms correction this effect was not significant anymore (*p* = 0.074). This could be due to a very subtle effect for which the Wilcoxon test is not powerful enough to detect. Also the small number of subjects should be taken into account.

## Discussion

The results of this clinical sonification study show that a musical sonification therapy may be a promising new way of treating motor impairments after stroke. Musical sonification therapy may even improve psychological well-being after stroke. The 15 patients of the musical sonification group improved significantly compared to the movement training group in the Fugl–Meyer subscale assessing joint pain. Reduced pain after a motor training or a mechano-acoustic vibration therapy for stroke patients was also shown by Lee and Kim (37) and Constantino et al. (38). The MG patients of our study also showed a trend to regain a better hand function in the SIS after the training. In addition to the motor domain, the SIS assesses the emotional state of the patient, memory, and social participation. Movement smoothness pre and post intervention was found to be significantly better after the therapy in the MG. This is in line with the findings of Rohrer et al. (21) who showed an increased movement smoothness with a robotic therapy device in four of five measures in 31 patients recovering from stroke. In contrast, the patients of our CG, receiving only a “sham” movement training without musical feedback improved very little and non-significantly in some of the tests. Thus, we assume that the musical aspect plays an important role in the sonification therapy. However, in this study, we did not control whether it is the musical aspect of sonification or just any sound information provided by the sonification. Furthermore, different motor tests should be included in future research in order to prevent floor (NHPT) and ceiling effects (ARAT), which were found in some of the tests in this study. Of course, as we only present a small clinical trial with limited statistical power, results need to be verified with a larger group of patients.

The novel aspect of our approach is that we encourage the patients in the musical sonification group to actively play and create music by moving their arms. This way, music was not only a byproduct of, e.g., a grasping motion. Instead, movements resembled more a novel musical instrument patients were starting to play. This musical instrument was sometimes compared to a theremin by professional musicians. Hence, our sonification training was designed to resemble a music lesson rather than shaping a movement during sound playback. Furthermore, we used musical stimuli, such as a musical major scale with discrete intervals and timbre parameters derived from the sound characteristics of acoustical musical instruments, as opposed to the widely used sound mappings where tone pitch is scaled continuously and rather artificial sounds are applied (39). The main idea underlying our hypothesis was that participants could improve control of arm positions in space *via* associative learning, leading to associating a given relative arm position with a specific musical sound. This sound-location association may then substitute the frequently declined, or even lost, proprioception. Additionally, the arm movement trajectories from outset to the target position were audible as well. Thus, multimodal learning might have taken place because patients received sound as an additional parameter supplying information. One could speculate that this multimodal learning could help to close the sensorimotor loop, which may have been affected by the stroke.

In view of the clinical application, reduced gross motor functions of the arm and reduced proprioception are common disabilities in stroke patients (40). Hence, the advantages of continuous real-time musical feedback are obvious: the therapy, therefore, aims at retraining gross motor movements of the arm, which are the most disabling challenges in early rehabilitation of stroke. Second, real-time sonification may substitute deficits in proprioception of the arm, which frequently are a consequence of stroke.

Finally, this form of therapy is highly motivating and could thus enhance motor functions and the emotional well-being in some patients, maybe through the creative, playful character of this musical sonification device (41–44).

To summarize, we have developed and tested a novel musical sonification therapy in a group of patients, which supports learning effects in auditory sensory–motor integration. Now, multimodal learning of spatial, motor, auditory, and proprioceptive information in rehabilitation of arm motor control in stroke patients needs to be evaluated in a larger multicentered representative randomized controlled clinical trial.

## Author Contributions

All authors listed have made substantial, direct, and intellectual contribution to the work and approved it for publication.

## Conflict of Interest Statement

The authors declare that the research was conducted in the absence of any commercial or financial relationships that could be construed as a potential conflict of interest.

## Footnotes

^1^http://www.r-project.org

^2^http://www.rstudio.com
